# Machine learning and analysis of genomic diversity of “*Candidatus* Liberibacter asiaticus” strains from 20 citrus production states in Mexico

**DOI:** 10.3389/fpls.2022.1052680

**Published:** 2022-12-15

**Authors:** Jiaquan Huang, Iobana Alanís-Martínez, Lucita Kumagai, Zehan Dai, Zheng Zheng, Adalberto A. Perez de Leon, Jianchi Chen, Xiaoling Deng

**Affiliations:** ^1^ Department of Plant Pathology, South China Agricultural University, Guangzhou, Guangdong, China; ^2^ Center for Biological Science and Technology, Advanced Institute of Natural Sciences, Beijing Normal University at Zhuhai, Zhuhai, Guangdong, China; ^3^ National Station of Plant Epidemiology, Quarantine and Sanitation, SENASICA, Queretaro, Mexico; ^4^ Plant Pest Diagnostic Center, California Department of Food and Agriculture, Sacramento, CA, United States; ^5^ United States Department of Agriculture-Agricultural Research Service (USDA-ARS), San Joaquín Valley Agricultural Sciences Center, Parlier, CA, United States

**Keywords:** “*Candidatus* Liberibacter asiaticus”, citrus HLB, mexico HLB, genomic diversity, machine learning, single nucleotide polymorphisms, sparse partial least squares discriminant analysis (sPLS-DA), principal component analysis (PCA)

## Abstract

**Background:**

Huanglongbing (HLB, yellow shoot disease) is a highly destructive citrus disease associated with a nonculturable bacterium, “*Candidatus* Liberibacter asiaticus” (CLas), which is transmitted by Asian citrus psyllid (ACP, *Diaphorina citri*). In Mexico, HLB was first reported in Tizimin, Yucatán, in 2009 and is now endemic in 351 municipalities of 25 states. Understanding the population diversity of CLas is critical for HLB management. Current CLas diversity research is exclusively based on analysis of the bacterial genome, which composed two regions, chromosome (> 1,000 genes) and prophage (about 40 genes).

**Methods and results:**

In this study, 40 CLas-infected ACP samples from 20 states in Mexico were collected. CLas was detected and confirmed by PCR assays. A prophage gene(*terL*)-based typing system (TTS) divided the Mexican CLas strains into two groups: Term-G including four strains from Yucatán and Chiapas, as well as strain psy62 from Florida, USA, and Term-A included all other 36 Mexican strains, as well as strain AHCA1 from California, USA. CLas diversity was further evaluated to include all chromosomal and prophage genes assisted by using machine learning (ML) tools to resolve multidimensional data handling issues. A Term-G strain (YTMX) and a Term-A strain (BCSMX) were sequenced and analyzed. The two Mexican genome sequences along with the CLas genome sequences available in GenBank were studied. An unsupervised ML was implemented through principal component analysis (PCA) on average nucleotide identities (ANIs) of CLas whole genome sequences; And a supervised ML was implemented through sparse partial least squares discriminant analysis (sPLS-DA) on single nucleotide polymorphisms (SNPs) of coding genes of CLas guided by the TTS. Two CLas Geno-groups, Geno-group 1 that extended Term-A and Geno-group 2 that extended Term-G, were established.

**Conclusions:**

This study concluded that: 1) there were at least two different introductions of CLas into Mexico; 2) CLas strains between Mexico and USA are closely related; and 3) The two Geno-groups provide the basis for future CLas subspecies research.

## Introduction

Huanglongbing (HLB, yellow shoot disease, also called citrus greening disease) is a highly destructive disease threatening the world citrus industry. HLB is associated with a nonculturable proteobacterium bacterium, “*Candidatus* Liberibacter asiaticus” (CLas). Under field conditions, CLas is transmitted by Asian citrus psyllid (ACP, *Diaphorina citri*). HLB was observed for over a hundred years in Asia (Bove, 2006; Lin, 1956), but was first reported in the United States in Florida in 2005 (Halbert, 2005), then in California in 2012 (Kumagai et al., 2013) and in Texas in 2012 (Kunta et al., 2012). In Mexico, the disease was first reported in Tizimin, Yucatán, in 2009 (Trujillo-Arriaga, 2011) and is now endemic in 351 municipalities of 25 states (SENASICA, 2022).

For effective HLB management, it is important to understand the population diversity of CLas strains and relationships among strains from different geographical sources. Since CLas cannot be cultured *in vitro*, strain characterizations are mostly derived from genomic sequence analyses. A CLas genome is about 1.2 Mbp and composed of a chromosomal region that hosts core genes, and a prophage region that contains dispensable genes (Duan et al., 2009; Zheng et al., 2014), and prophage could be absent (Katoh et al., 2014). The CLas prophage region is highly variable and has been the target in CLas diversity studies (Chen et al., 2010; Liu et al., 2011; Deng et al., 2014; Zheng et al., 2017; Alanís-Martínez et al., 2018; Dai et al., 2019; Fu et al., 2020; Zheng et al., 2021). Based on single nucleotide polymorphisms (SNPs) in a 370-bp region in *terL*, a gene encoding a phage terminase large subunit, the *terL* typing system (TTS) was proposed (Deng et al., 2014). In contrast, due to the highly conserved nature of the CLas genome, few chromosomal loci have been found to be effective for CLas diversity research (Bastianel et al., 2005; Deng et al., 2008). The exception was a tandem repeat locus located in chromosomal region annotated as a gene encoding for a bacteriophage repressor protein (Chen et al., 2010; Katoh et al., 2011; Ma et al., 2014; Ghosh et al., 2015; Singh et al., 2019). Analyses on both prophage and chromosomal regions are needed for a complete evaluation of CLas genomic diversity. Since TTS is prophage-based, use of chromosomal gene(s) that support TTS will strengthen CLas diversity research.

Thanks to the development of next-generation sequencing (NGS) technology, there are currently 39 whole genome sequences of CLas with various completion status (either complete with a single contig or draft with multiple contigs) in GenBank database (version 249). Eighteen CLas genome sequences were from the United States (9 from California, 5 from Florida, and 6 from Texas) (**Supplementary Table 1**). In contrast, there is only one Mexican CLas genome sequence (Mex8, related to Texas and Florida strains) published (Thapa et al., 2020). More CLas genome sequences from Mexico are in need for CLas diversity research.

Genome sequence analyses involve data sets of Mbp or Gbp that generate multi-dimensional data outputs, i.e., data matrices with a large number of columns and rows. Handling and interpreting such complex data sets are highly challenging through conventional approaches. Machine learning (ML) is a technology that uses computational statistics to resolve complex data problems. In general, there are two types of ML. Unsupervised ML takes a high-dimensional set of data to find or predict structures such as clusters, e.g., principal component analysis (PCA) (Abdi and Williams, 2010) Supervised ML builds a mathematical model of a set of data that contains guidance to generate desired outputs, e.g., sparse partial least-squares discriminant analysis (sPLS-DA) (Lê Cao et al., 2011; Lee et al., 2018).

The goal of this study was to characterize the genomic diversity of CLas strains from Mexico. The information was used to evaluate strain evolutionary paths and to reveal relationships of CLas strains within Mexico and between Mexico and the neighboring USA. CLas strains were collected from 20 states in Mexico and typed by TTS. Representative strains were sequenced. CLas diversity were evaluated using ML approaches to resolve research issues associated with data processing and result interpretations. Two Geno-groups of CLas were established. Potential impacts of the Geno-groups on HLB epidemiology and CLas biological research were discussed.

## Materials and methods

### CLas strain DNA collection

The ACP samples from 20 states in Mexico were collected in the period from September, 2017 to August, 2018 from commercial citrus orchards and backyard trees in urban areas (**Table 1**). The trees where the psyllids were collected were *Citrus aurantiifolia*, *C. sinensis*, *C. latifolia*, *C. reticulata*, as well as the ornamental species *Murraya paniculata*. Each ACP sample contained 1 to 50 insects preserved in 70% ethanol. DNA was obtained using the E.Z.N.A.^®^ Tissue DNA Kit (Omega Bio-Tek, D3396-02) according to the manufacturer’s instructions.

**Table 1:** General information of Asian citrus psyllid (ACP, *Diaphorina citr*i) samples collected from 20 states in Mexico and the result of PCR detections and typing with *terL* typing system (TTS).

No.	Geographic origin	HLBas/HLBr	CLas-G/ HLBr	RNRf/RNRr	TTS^a^	Citrus species	Collection year
1	Baja California Sur	24.22	21.68	20.72	A	*C. sinensis*	Jan. 2018
2	Baja California Sur	26.49	23.19	22.06	A	*C. sinensis*	Jan. 2018
3	Chiapas	32.49	29.01	28.25	G	*C. latifolia*	Mar. 2018
4	Chiapas	25.61	27.46	27.33	G	*C. latifolia*	Mar. 2018
5	Colima	25.71	27.97	27.01	A	*C. aurantiifolia*	Jun. 2018
6	Colima	26.89	23.32	22.65	A	*C. aurantiifolia*	Aug. 2018
7	Guerrero	32.31	27.67	26.82	A	*C. aurantiifolia*	Jan. 2018
8	Guerrero	25.25	23.22	22.62	A	*C. aurantiifolia*	Jan. 2018
9	Hidalgo	33.33	36.99	36.89	ND	*C. sinensis*	Oct. 2017
10	Hidalgo	26.73	24.14	23.32	A	C. spp; *Murraya paniculata*	Dec. 2017
11	Jalisco	34.5	31.45	29.99	A	*C. latifolia*	Jan. 2018
12	Jalisco	24.23	22.65	22.04	A	*C. latifolia*	Mar. 2018
13	Michoacan	19.16	22.75	22.94	A	*C. aurantiifolia*	Oct. 2017
14	Michoacan	21.4	24.51	23.87	A	*C. aurantiifolia*	Oct. 2017
15	Morelos	24.61	20.96	20.19	A	*C. sinensis*	Jan. 2018
16	Morelos	23.24	20.36	19.8	A	*C. aurantiifolia*	Jan. 2018
17	Nayarit	20.64	18.24	17.01	A	*C. latifolia*	Jan. 2018
18	Nayarit	25.66	28.54	26.77	A	*C. latifolia*	Jan. 2018
19	Nuevo Leon	29.14	25.69	24.95	A	Citrus spp	Dec. 2017
20	Nuevo Leon	27.59	26.88	26.26	A	*C. aurantiifolia*	Jul. 2018
21	Oaxaca	33.24	28.5	27.48	A	*C. latifolia*	Oct. 2017
22	Oaxaca	27.52	24.46	23.69	A	*C. aurantiifolia*	Dec. 2017
23	Puebla	26.13	26.47	25.4	A	*C. sinensis*	Mar. 2018
24	Puebla	25.66	26.86	26.03	A	*C. sinensis*	Mar. 2018
25	Queretaro	25.23	22.48	22.91	A	*C. sinensis*	Jan. 2018
26	Queretaro	27.2	24.55	23.88	A	*C. sinensis*	Jan. 2018
27	San Luis Potosi	33.83	29.32	28.27	A	*C. sinensis*	Dec. 2017
28	San Luis Potosi	29.38	26.23	25.27	A	*C. sinensis*	Mar. 2018
29	Sinaloa	29.66	30.11	29.09	A	*C. aurantiifolia*	Aug. 2018
30	Sinaloa	27.93	28.54	27.63	A	*C. aurantiifolia*	Aug. 2018
31	Sonora	33.36	31.7	30.72	A	*C. aurantiifolia*	Dec. 2017
32	Sonora	31.69	31.71	31.98	A	*C. aurantiifolia*	Dec. 2017
33	Tamaulipas	29.48	25.23	24.29	A	*C. sinensis*	Dec. 2017
34	Tamaulipas	27.14	22.99	21.81	A	*C. sinensis*	Dec. 2017
35	Veracruz	25.21	23.71	22.68	A	*C. sinensis*	Sep. 2017
36	Veracruz	26.88	23.99	23.14	A	*C. reticulata*	Sep. 017
37	Yucatan	19.26	19.29	18.23	G	*Citrus* spp.	Feb. 2018
38	Yucatan	25.17	24.58	23.81	G	*Citrus* spp.	Feb. 2018
39	Zacatecas	30.56	29.29	28.95	A	*C. latifolia*	May 2018
40	Zacatecas	27.67	25.6	24.88	A	*C. latifolia*	Aug. 2018

### CLas PCR

CLas in a sample was determined by three quantitative PCR assays. 1) TaqMan qPCR with primer set HLBas/HLBr and probe HLBp (HLBas-PCR, Li et al., 2006) performed in Mexico; 2) TaqMan PCR with primer set CLas-4G/HLBr and probe HLBp (CLas-4G-PCR, Bao et al., 2020); and TaqMan PCR with primer set RNRf-RNRr and probe RNRp (RNRf-PCR, Zheng et al., 2016) performed in California, USA.

HLBas-PCR (Li et al., 2006) was used as a primary detection. PCR was carried out in the CFX96 thermocycler (Bio-Rad Laboratories) in a volume of 25 µl with the following reagents: 1U Platinum Taq DNA polymerase (Invitrogen, USA), 1× Buffer PCR, 6 mM MgCl_2_, 0.2 mM dNTPs, 240 nM of each primer, 120 nM of target probe, and 2 µl DNA template. PCR amplification started at 95°C for 20 seconds, followed by 40 cycles of 95°C for 1 second and 58°C for 40 seconds. The fluorescence signal was captured at the end of each 58°C step. The data were generated from CFX96 Manager software with default parameters.

CLas-4G-PCR and RNRf-PCR were carried out as confirmatory tests. PCR was performed in Applied Biosystems (ABI) StepOnePlus Real-time PCR system. A reaction mixture (20μl) contained 10 μl of TaqMan^®^ Fast Universal PCR Master Mix (2X) (Applied Biosystems, ABI), 1 μl of DNA template (25 ng), 0.2 μl of TaqMan^®^ probe (5 μM), 0.4 μl of each forward and reverse primer (10 μM). PCR amplification started at 95°C for 20 seconds, followed by 40 cycles of 95°C for 10 seconds and 60°C for 20 seconds. The fluorescence signal was captured at the end of each 60°C step. StepOnePlusTM Software v2.3 (Applied Biosystems) was used to analyze the data, with automated baseline settings and a manually set threshold of 0.1.

### Strain typing using TTS

For PCR typing, the procedure described previously (Deng et al., 2014) was followed. Primer set CT3f/CT3r (5’ CCAGAAACGA TTAGC GTTCT GAT 3’/5’ GTCAATAAGA CCTGGTATGT CTA 3’) was used for Term-A type, and primer set FC3f/FC3r (5’ CCAGAAACGA TTAGCGTTCC ACT 3’/5’ CGACCAGGGA TATCGG 3’) was used for Term-G typing.

For sequencing typing, CLas DNA samples were amplified by conventional PCR using primer set 766-f/766-r (5’ CAATAACAGG ACCTCTACGC 3’/5’ ATTGGAAAGA CGACGTTAAA 3’) (Liu et al., 2011) with the following steps: initial denaturation for 3 min at 95°C, followed by 35 cycles of denaturation for 45 seconds at 95°C, annealing for 30 seconds at 55°C, elongation for 2 min 30 seconds at 72°C, and a final extension step of 72°C for 10 min. The amplified DNA fragments were collected from NucleoSpin^®^ Gel and purified by PCR Clean-up kit (QIAGEN, Valencia, USA) following the manufacturer’s protocol, and sequenced using ABI BigDye Terminator Chemistry (Life Technologies) in an ABI 3730 sequencer (Life Technologies) with 766-f/766-r. The *terL*-370 bp segment, including the CT3f/CT3r and FC3f/FC3r regions, was identified and extracted from each amplicon sequence. All sequences were aligned using CLUSTAL program (Thompson et al., 1994). Single nucleotide polymorphisms (SNPs) were identified manually for assignment of Term-A or Term-G group.

### Whole genome sequencing and assembling

Two samples, YTMX (Term-G) from Yucatán (No. 1 in **Table 1**) and BCSMX (Term-A) from Baja California Sur (No. 37 in **Table 1**) were selected for whole genome sequencing following the procedure described previously (Zheng et al., 2014; Huang et al., 2021). Briefly, 2–4 µl of sample DNA (mixture of bacteria + ACP) was enlarged (i.e., increased all DNA simultaneously) with GenomiPhiTM V2 DNA Amplification kit (GE Healthcare, Sigma-Aldrich Corp., St. Louis, MO, USA) following manufacturer’s instructions. The enlarged DNAs were sequenced by Illumina HiSeq (2x100) formats through commercial sources. Only sequence reads with Q score > 30 were collected. Quality check of the Illumina reads was performed using FastQC (Andrews, 2010). The HiSeq reads were assembled using reference-mapping with the whole genome sequences of psy62 (NC_012985.3), A4 (NZ_CP010804.2) and AHCA1 (NZ_CP029348.1) using CLC Genomic Workbench 10.0 with the parameters: Length Fraction = 1 and Similarity Fraction = 0.9. *de novo* assembly was also conducted using default parameters. A final complete genome sequence was obtained by combining the assemblies of reference-mapping and *de novo* assembly. The genome sequence annotations were performed using Prokka v1.14.6 (Seemann, 2014).

### Unsupervised ML

All 39 CLas genome sequences deposited in GenBank database (version 249) were downloaded (**Supplementary Table 1**). One representative sequence each from “*Ca.* L. africanus” (CLaf), “*Ca*. L. americanus” (CLam), and “*Ca*. L. solanacearum” (CLso) were also downloaded. Strain Tabriz.3 showed average nucleotide identity (ANI) value lower than 95 and (**Supplementary Table 2**), therefore not considered as CLas in this study. The genome sequences HHCA (JMIL02), SGpsy (QFZJ01), and SGCA1 (QFZT01) that had few contigs longer than 1,000 bp were excluded for ANI calculation. The 35 (39 – 4) CLas strain sequences along with the BCSMX and YTMX genome sequences, adding to a total of 37 CLas genome sequences, and the 3 non-CLas genome sequences (1 CLaf, 1 CLam, and 1 CLso) were used for pair-wise ANI calculation using FastANI v1.33 (Jain et al., 2018) with fragment size set to 1,000 bp. Relationships among CLas strains embedded in the ANI matrix were revealed through PCA with mixOmics R package (v6.10.8) (Rohart et al., 2017). The TTS typing of each genome was implemented through BLAST search with the *terL*-370-bp sequence in reference to SNPs matching CT3f/CT3r or FC3f/FC3r. If no SNP match, the genome was labeled as non-typeable.

### Supervised ML

The label of Term-A or Term-G type of a genome was used as a guide to identify genes with supportive SNPs. Details procedures were listed in **Table 2**. Briefly, 1) With the exception of the non-TTS typeable strain Ishi-1 (NZ_AP014595.1), 10 CLas whole genome sequences with a single contig (“complete” status), and the two Mexican CLas genome sequences from this study were selected (**Supplementary Table 1**). The genome sequences were reannotated to generate uniform feature format (.gff) file; 2) Single-copy core genes were identified using Panaroo v1.2.10 software (Tonkin-Hill et al., 2020); 3) SNPs in the core gene were identified using MAFFT v7.490 software (Katoh and Standley, 2013) and SNP-sites v2.5.1 (Page et al., 2016); 4). Based on TTS, each genome was labeled as either Term-A or Term-G for parameters tuning; and 5) sPLS-DA was performed with the mixOmics R package (v6.10.8) to identify genes with supportive SNPs for TTS. The features with contribution values to each component >= |0.2| were selected. Each SNPs in the two Mexican genomes were confirmed by strict read mapping performed with CLC Genomic Workbench 10.0 (Length Fraction = 1; Similarity Fraction = 0.97).

**Table 2:** A workflow of sparse partial least-squares discriminant analysis (sPLS-DA) in this study.

Step	Inputs	Outputs	Process	Software
1	CLas genome	A .gff file	Reannotated genome and acquired .gff files	Prokka
2	A .gff file	Shared genes	Acquired shared genes of 12 selected CLas genome	Panaroo
3	Shared genes	SNPs matrix	Generated SNPs matrix from shared genes of 12 complete CLas genome	Mafft, SNP-sites, custom scripts
4	SNPs matrix, Labels (Term-A, Term-G), default parameter	Estimated counts of component and its attributed features (Optimized parameter)	Parameters tuning and numerical outputs	*perf* and *tune.splsda* function in mixOmics R package (v6.10.8)
5	SNPs matrix, Labels (Term-A, Term-G), optimized parameter	Suitable features for best performance on classification	Getting the genes features that support the Term-A and Term-G group.	*splsda* and *plotIndiv* in mixOmics R package (v6.10.8)

Using the genome sequence of strain AHCA1 (NZ_CP029348.1) as a reference, all genes with TTS supportive SNPs were identified. For each TTS SNP, a 51-bp sequence with the SNP in the center (nucleotide position 21) was copied. The 51-bp sequence was used as a query to BLAST search against the 40 CLas genome sequences (38 sequences in GenBank excluding strain Tabriz.3 and two Mexican strain genomes in this study, **Supplementary Table 1**). TTS SNPs in each genome were identified manually. TTS SNPs in each CLas genome were concatenated for cluster analysis using ggdendro and ggplot2 (Wickham, 2016).

## Results

**Figure 1:**
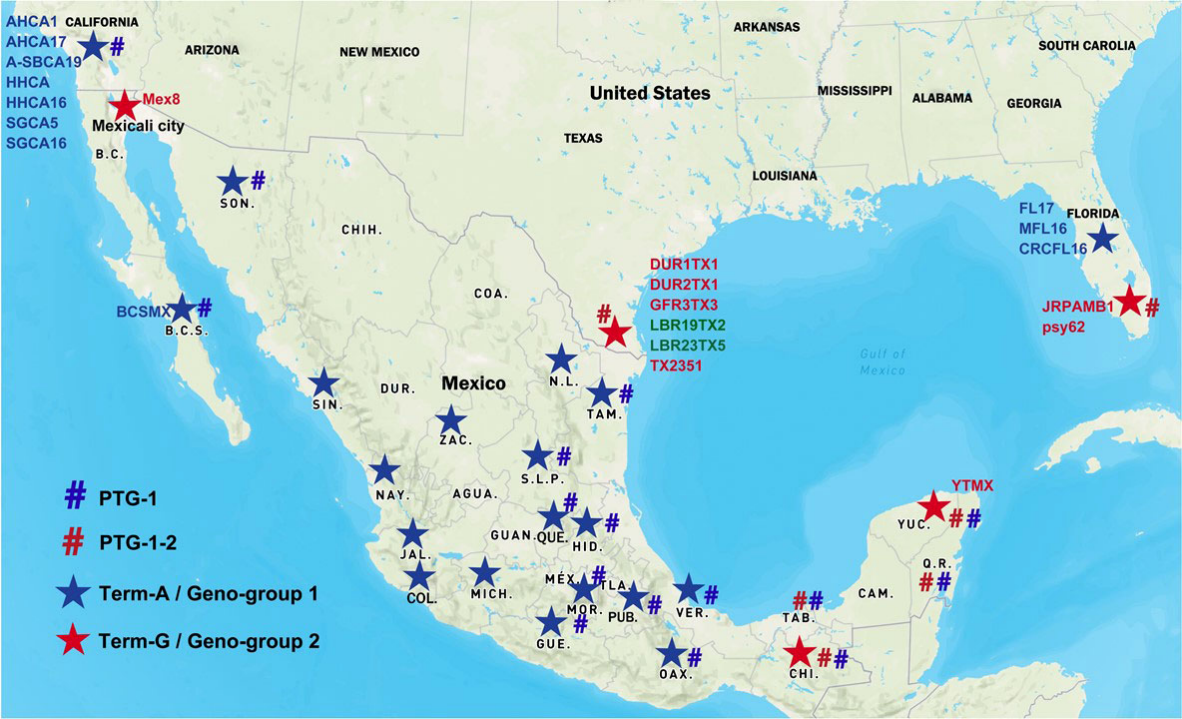
Distribution of “*Candidatus* Liberibacter asiaticus” (CLas) strains in Mexico and the United States. Blue star signs represent Term-A group/Geno-group 1; Red star signs represent Term-G group/Geno-group 2. Blue number signs represent PTG-1 prophage group; Red number signs represent PTG-1-2 prophage group. Two CLas strains in green were non-typeable for neither Term-A or Term-G, but determined to be in Geno-group 2.

A total of 40 Asian citrus psyllid (ACP) samples were collected from 20 states in Mexico (**Table 1** and **Figure 1**). The HLBas-PCR Ct values ranged from 19.16 to 34.50. CLas was further confirmed by CLas4G-PCR despite of the variations of Ct value ranges in some samples. In all samples, Ct values of RNRf-PCR were lower than those of CLas4G-PCR, a further validation of CLas detection (Bao et al., 2020). Of particular interest was sample #9 from Hidalgo with a Ct of 36.99 by CLas4G-PCR. Such a high Ct value could have been attributed to the presence of non-CLas bacteria (Huang et al., 2021). However, when sample #9 was cross-checked using the more sensitive RNRf-PCR, a lower Ct value confirmed the presence of CLas (Bao et al., 2020).

TTS by PCR grouped the Mexican CLas strains into Term-A and Term-G groups (**Table 1** and **Figure 1**). Term-G included strains in the states of Yucatán and Chiapas in Peninsula and southern Mexico, and strain psy62, the major CLas strain in Florida, USA. Term-A included the other 36 strains from central and northern Mexico, and strain AHCA1 reported in California, USA (Dai et al., 2019). Analyses of amplicon sequences confirmed the TTS typing results with the exception of sample #9, where no amplicon was available for sequencing. Interestingly, the Mexicali strain in the central and northern Mexico was in Term-G group (**Figure 1**).

Selected metrics of whole genome sequences of CLas strains YTMX and BCSMX are summarized in **Table 3**. HiSeq sequencing generated a total of 492,619,792 reads (49,754,598,992 bp, ~50 G bp) for YTMX. HiSeq reads mapped the best to psy62 with the largest number of 625,260 reads, as comparing to A4 (623,691 reads) and AHCA1 (622,913 reads). Therefore, psy62 was used as the main reference for YTMX genome assembling. The CLas strain YTMX genome was 1,229,040 bp in 4 contigs with three prophages. The circular form of all three prophages was detected. Similarly, a total of 571,293,176 reads (57,700,610,776 bp, ~58 G bp) for strain BCSMX. HiSeq reads mapped the best to AHCA1 (141,652 reads), comparing to psy62 (140,531 reads) and A4 (137,757 reads). Therefore, AHCA1 was used as the main reference for BCSMX genome assembling. The BCSMX genome was 1,230,223 bp in 4 contigs with one prophage and the circular form was detected.

**Table 3:** Selected metrics of two whole genome sequences of “*Candidatus* Liberibacter asiaticus” strains from Mexico.

Strain	Genome region	Contig numbers	Genome size (bp)	G+C (%)	Open Reading Frame	rRNA genes	tRNA genes
YTMX	Chromosome	4	1,229,040	36.5	1186	9	44
	Type 1 phage	1	40067	41.2	40	0	0
	Type 2 phage	1	38988	39.4	47	0	0
	CLasMV1	1	8867	36.7	11	0	0
BCSMX	Chromosome	4	1,230,223	36.5	1187	9	44
	Type 1 phage	1	38064	41.9	40	0	0

**Figure 2:**
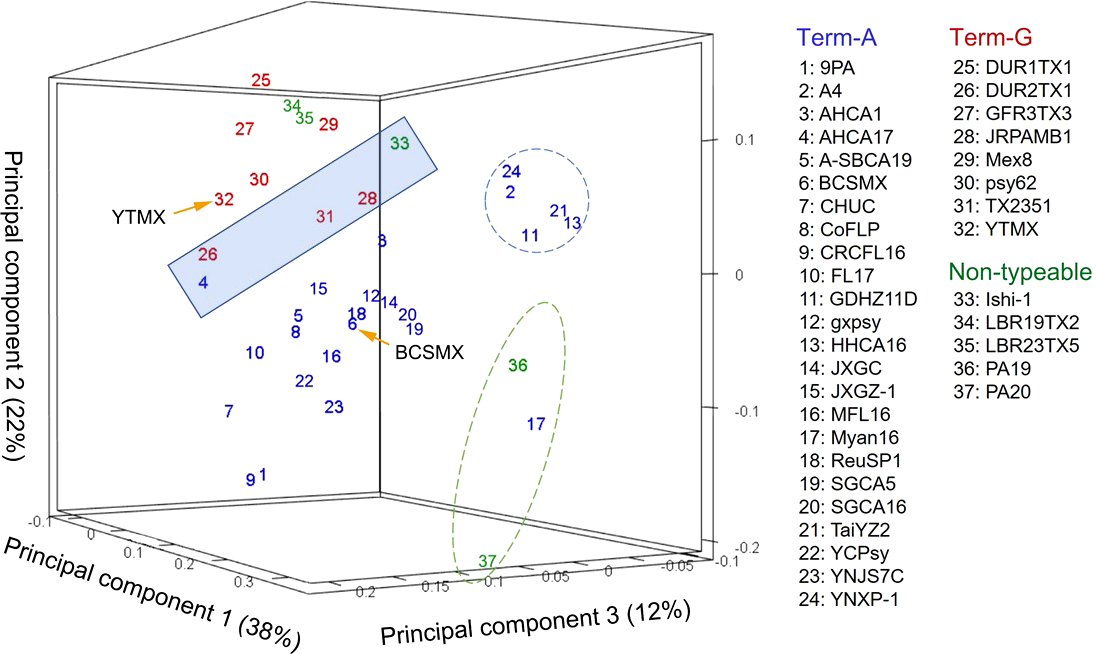
Relationships of “*Candidatus* Liberibacter asiaticus” (CLas) strains revealed by an unsupervised marchine learning (ML) tool (principal component analysis) on average nucleotide identity (ANI) values. Each strain is represented by a number with the correspondent names listed on the right. Blue numbers represent Term-A group; Red numbers represents Term-G group; and green numbers represents non-typable for Term-A or Term-G. Note that strains in the shaded box borderline between Term-A and Term-G groups. The grouping status of the borderline strains as well as the non-typeable strains was resolved/confirmed in a supervised ML approach (**Figure 3**). Although not the objective of this study, it was interesting to note that strains in blue circle harbored Type 2 prophage only, and strains in green circle were of South Asia origins (#17 from Myanmar; #36 and #37 from Pakistan).

In the unsupervised machine learning (ML) study, pair-wise average nucleotide identity (ANI) values of the 37 CLas strains ranged from 99.67 to 100.00 (**Supplementary Table 2**), indicating the high level of genome sequence congruence of CLas strains. The ANIs of all CLas strains to the non-CLas strains were significantly lower with CLaf at 76.21, CLam at 75.91, and CLso at 76.72. Results of PCA analysis on the CLas strain matrix (**Supplementary Table 2**) are presented in **Figure 2**. The three PCAs accounted for 72% of the total variance. Term-G strains (in red) are separated from Term-A strains (in blue). For the five non-typeable strains (in green), strains LBR19TX2 (#34) and LBR23TX5 (#35) were grouped with Term-G, so was strain Ishi-1 (#33). Strain PA19 (#36) and PA20 (#37) (green dash-line circle) appeared to be distant from Term-A and Term-G groups, along with the Term-A strain Myan16 (#17). It is also interested that strains circled by blue dash-line, TaiYZ2 (#21), YNXP-1(#24), A4 (#2), HHCA16 (#13), GDHZ11D(#11), harbored Type 2 prophage only.

In the supervised ML study, analysis of the 12 selected CLas genomes identified a total of 1,044 single-copy core genes. Sparse partial least squares discriminant analysis (sPLS-DA) identified 15 *terL* typing system (TTS) supportive genes, each having 1 to 5 SNPs. A closer examination found that some SNPs could subgroup within Term-A and Term-G groups. SNPs supporting TTS are shown in **Table 4**, along with their correspondent positions in the reference genomes (AHCA1, NZ_CP029348.1 and psy62, NZ_CP010804.2). Gene 1 to 14 were from the chromosomal region and Gene 15 was from the prophage region (**Table 4**). The length of these genes varied from 183 to 5,487 bp according to the annotation of CLas strain AHCA1 and psy62 (**Table 4**).

**Table 4:** List of genes with single nucleotide polymorphisms (SNPs) supporting the *terL* typing system (TTS) based on partial least squares discriminant analysis (sPLS-DA) on 12 selected genomes sequences of “*Candidatus* Liberibacter asiaticus” strains (column A to L**^a^**).

Gene No.	Annotation	Gene size (bp)	Total SNPcount	TTS SNP	A (AHCA1 genome position)	B	C	D	E	F	G	H	I	J(psy62 genome position)	K	L
1	Hypothetical protein	294	1	1	C (101583)	C	C	C	C	C	C	C	C	A (107799)	A	A
2	DNA topoisomerase IV subunit A (parC)	2262	2	1	G (244215)	G	G	G	G	G	G	G	G	C (250429)	C	C
3	Von Willebrand factor type A domain	1377	1	1	C (282667)	C	C	C	C	C	C	C	C	T (288883)	T	T
4	DNA mismatch repair protein (mutS)	2763	1	1	G (515669)	G	G	G	G	G	G	G	G	A (521855)	A	A
5	Signal peptide peptidase (sppA)	882	4	1	A (518650)	A	A	A	A	A	A	A	A	C (524835)	C	C
6	Flp family type IVb pilin	183	5	3	A (527597) G (527663) C (527697)	A G C	A G C	A G C	A G C	A G C	A G C	A G C	A G C	C (533784) A (533851) T (533884)	C A T	C A T
7	Fructose-bisphosphate aldolase, class I	1020	1	1	C (614670)	C	C	C	C	C	C	C	C	T (620849)	T	T
8	DUF4167 domain-containing protein	633	2	1	G (842355)	G	G	G	G	G	G	G	G	A (848572)	A	A
9	IS1595 family transposase	534	1	1	C (947957)	C	C	C	C	C	C	C	C	T (954187)	T	T
10	Homoserine/homoserine lactone efflux protein	609	1	1	T (974537)	T	T	T	T	T	T	T	T	C (980766)	C	C
11	N-acetyltransferase	558	1	1	G (980940)	G	G	G	G	G	G	G	G	A (985061)	A	A
12	Excinuclease ABC subunit (uvrB)	2418	2	1	C (984688)	C	C	C	C	C	C	C	C	T (988809)	T	T
13	Chemotaxis sensory transducer	5487	1	1	G (1027292)	G	G	G	G	G	G	G	G	A (1031393)	A	A
14	Hypothetical protein	591	3	3	G (1140678) C (1140556) G (1140532)	G C G	G C G	G C G	G C G	G C G	G C G	G C G	G C G	T (1140213) T (1140090) T (1140060)	T T T	T T T
15	Phage DNA polymerase	2028	3	2	T (1231071) A (1231378)	T A	T A	T A	T A	T A	T A	T A	T A	G (4155) G (4462)	G G	G G

^a^ A, AHCA1 (California); B, A4 (China); C, TaiYZ2 (Tailand); D, GDHZ11D (China); E, BCSMX (Mexico); F, CoFLP; (Columbia); G, ReuSP1 (Africa); H, JXGC (China); I, YCPsy (China); J, YTMX (Mexico); K, psy62 (Florida); L, JRPAMB1 (Florida).

**Figure 3:**
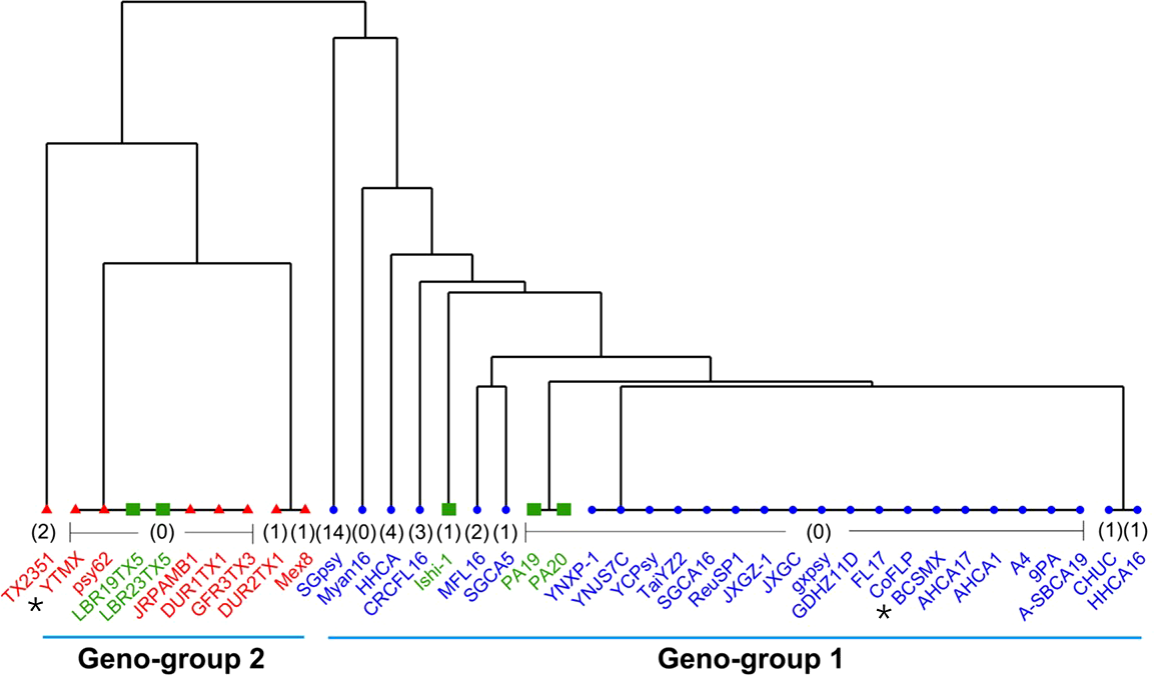
A dendrogram showing the Geno-group status of “*Candidatus* Liberibacter asiaticus” (CLas) strains with whole genome sequences available in GenBank based on concatenated SNPs from 15 TTS (*terL* typing system) supportive genes. Strains in blue are in Term-A group; Strain in red are in Term-G group; and strains in green are non-typeable for Term-A or Term-G. The number in bracket indicates absent SNPs of that strain due to the incomplete nature of the draft genome sequence. The two Mexican CLas strains in this study are indicated by *.

The TTS SNPs identified through BLAST search against the 40 CLas genomes (12 complete and 28 draft) sequences are summarized in **Supplementary Table 3**. Because of the incomplete status, not all TSS SNPs were found in each draft genomes. A dendrogram based on the concatenated SNPs (**Supplementary Table 3**) is shown in **Figure 3**. The Term-A and Term-G clusters are clearly defined. For the non-typeable strains, LBR19TX2 and LBR23TX5 fall into the Term-G group; strains PA19 and PA20 formed a unique cluster within Term-A group, and strain Ishi-1 is within the Term-A group.

## Discussions

An important contribution of this study was the identification of chromosomal core genes with SNPs for typing CLas strains from Mexico, as well as from other geographical regions (**Table 4** and **Supplementary Table 3**). This was achieved by the analyses of CLas whole genome sequences using ML approaches. While the prophage-based TTS (*terL* typing system) has shown its capacity for CLas strain typing (Deng et al., 2014), there was no support from chromosomal genes until this study. Theoretically, each chromosomal gene can be analyzed one by one to identify TTS SNPs. This is apparently not practical for >1,000 genes in a CLas genome. The unsupervised ML (PCA) served as a first step to show the pattern of CLas strain clusters at the whole genome sequence level (**Figure 2**). Some strains fall in the border region between Term-A and Term-G groups and some strains were not typeable by TSS. The supervised ML (sPLS-DA) defined the CLas groups with SNPs in 14 core genes along with a prophage gene and resolved the CLas strain typing issue (**Figure 3**). Putting together all the evidence, two CLas genomic groups, designated as Geno-groups, are proposed: Geno-group 1 that extends the Term-A group, and Geno-group 2 that extends the Term-G group.

The two distinct Geno-groups in Mexico set the foundation for epidemiological analysis. First, it suggests that there were at least two different introductions of CLas in Mexico. The exact timelines and routes of introduction could not be determined with the current information. It is, however, clear that CLas strains in Mexico were related to those in the US (**Figure 1**). It is generally believed that long distant CLas spread is through infected propagative materials along with ACPs. The original source of CLas and ACP in Mexico is currently not clear (Fuentes et al., 2018). Mexico allows importation of citrus propagative material (buds) from the US and Spain. Although strict quarantine procedures were enforced through the Mexico Citrus Propagation Material Certification Program, CLas was not in the scope of a regulation until 2005 when HLB was detected in Florida. Furthermore, one cannot rule out illicit host material brought or shipped in as a source of introduction. However, it should be noted that for a better understanding of CLas introduction and spread, much more information, including ACP diversity and records of citrus material movements are needed.

With the detection of CLas (now confirmed as Geno-group 2) in Yucatan in 2009, the Mexico federal government implemented various phytosanitary actions to limit the spread of the disease. Through regulatory mechanisms, the movement of citrus plants within the country was restricted. The efforts appeared to be effective since Geno-group 2 is still restricted in the Peninsula states (**Figure 1**). However, there was evidence that Geno-group 1 strains spread quickly from west to east in the central and north of Mexico (**Figure 1**). CLas was recorded in the states of the Pacific Ocean coastal zone between December, 2009 and July, 2010, in the central states from April, 2011 to September, 2013, and finally in the states located in the Gulf of Mexico in December, 2015. The rapid dispersion of CLas from the Pacific coast to other areas has probably been favored by the prevalence of lemon plantations, whose almost continuous vegetative sprouting for most of the year favors the reproduction of the ACP (Robles-González et al., 2018).

The whole genome sequencing efforts reveal that both strains BCSMX and YTMX harbor prophages/phages (**Table 3**). BCSMX has only one Type 1 phage, and therefore belongs in the prophage typing group (PTG-1) that was also found in California (Dai et al., 2019). Strain YTMX contained Type 1 and Type 2 phages (PTG-1-2) like those from Florida (Zhang et al., 2011) and Texas (Kunta et al., 2012). This supports the finding of two different prophage types of CLas, PTG-1-2 in Peninsula and South of Mexico and PTG1 in Central and Northern Mexico (**Figure 1**) (Alanís-Martínez et al., 2018; Martínez-Carrillo et al., 2019).

In addition, CLasMV1, a single-stranded DNA phage recently reported in China (Zhang et al., 2021), was found in strain YTMX. This is the first report of CLasMV1 outside China, although fractions of CLasMV1 sequences (but not the complete phage genome) could be found in some published CLas genomes (Zhang et al., 2021). The CLasMV1 sequence from YTMX was highly similar to those described in the Chinese CLas strains (34 gaps, 11 SNPs, data not shown). No CLasMV1 type of phage was found in strain BCSMX. The different status of CLasMV1 between strains YTMX and BCSMX adds further evidence to support the two CLas Geno-groups in Mexico.

The establishment of two CLas Geno-groups encourages CLas subspecies research. Intra-specific diversity information is important for both CLas biology research and HLB management. There have not been reports on subspecies in CLas, despite the many efforts of strain diversity investigations. Multiple subspecies have been proposed for CLaf, the pathogen of the African form HLB (Garnier et al., 2000; Roberts et al., 2015; Roberts and Pietersen, 2017). Along this line, it is also noted that, based on PCA results (**Figure 2**) and core gene SNPs (**Figure 3**), the non-TTS typeable strains, PA19 and PA20, from Pakistan (Cui et al., 2021) could represent a new Geno-group although more studies are needed in the future.

## Conclusion

This study characterized the genomic diversity of CLas strains from Mexico using ML assisted genomic approaches. Two CLas Geno-groups were established. Research results suggested that 1) there were at least two different introductions of CLas into Mexico; 2) CLas strains between Mexico and USA are closely related; and 3) The two Geno-groups provides a base for future CLas subspecies study.

## Data availability statement

The datasets presented in this study can be found in online repositories. The names of the repository/repositories and accession number(s) can be found in the article/**Supplementary Material**.

## Author contributions

JC, XD, and JH designed the project, conducted the data analysis, and wrote the manuscript. IA-M, LK and AP performed the samples collection and facilitate sample and data processing. ZD and ZZ assisted in data analyses. All authors contributed to the article and approved the submitted version.

## Supplementary material

Supplementary Table 1 General information of “*Candidatus* Liberibacter” genome sequences available in GenBank database (version 249)

Supplementary Table 3 List of single nucleotide polymorphisms (SNPs) in genes supporting the *terL* typing system (TTS) from 26 draft genomes sequences of “*Candidatus* Liberibacter asiaticus” strains with Strain AHCA1 and Psy62 as references highlighted in yellow.
